# New Directions for the Treatment of Adrenal Insufficiency

**DOI:** 10.3389/fendo.2015.00070

**Published:** 2015-05-06

**Authors:** Gerard Ruiz-Babot, Irene Hadjidemetriou, Peter James King, Leonardo Guasti

**Affiliations:** ^1^Centre for Endocrinology, William Harvey Research Institute, Barts and the London School of Medicine and Dentistry, Queen Mary University of London, London, UK

**Keywords:** regeneration, adrenal cortex, stem cells, transplantation, encapsulation, steroidogenesis, zonation, SF1

## Abstract

Adrenal disease, whether primary, caused by defects in the hypothalamic–pituitary–adrenal (HPA) axis, or secondary, caused by defects outside the HPA axis, usually results in adrenal insufficiency, which requires lifelong daily replacement of corticosteroids. However, this kind of therapy is far from ideal as physiological demand for steroids varies considerably throughout the day and increases during periods of stress. The development of alternative curative strategies is therefore needed. In this review, we describe the latest technologies aimed at either isolating or generating *de novo* cells that could be used for novel, regenerative medicine application in the adrenocortical field.

The adrenal cortex is the primary site of steroid synthesis, producing glucocorticoids under the control of the hypothalamic–pituitary–adrenal (HPA) axis and mineralocorticoids under the control of the renin–angiotensin system. Glucocorticoids affect carbohydrate metabolism and mediate the mammalian stress response, while mineralocorticoids control blood volume and salt homeostasis. For this reason, the adrenal cortex is essential for life.

The adrenal cortex originates in a group of mesoderm-derived cells lying between the urogenital ridge and the dorsal aorta, forming the precursors of the adrenal glands and the gonads, the adrenogonadal primordium (AGP) (1). At around embryonic day (e)9.0 in the mouse, these cells begin to express the transcription factor steroidogenic factor 1 (SF1), which is essential for both adrenal and gonadal development (2). SF1 not only binds to response elements in the promoter regions of steroidogenic genes to positively regulate their transcription but can also be considered a true effector of cell fate as it starts a genetic program driving embryonic mesenchymal cells toward a steroidogenic phenotype/lineage (3, 4); its absolute requirement for steroidogenesis has been recently demonstrated *in vivo* (5). Other than SF1, at least four additional transcription factors have been shown to be key determinants of adrenal cortex development: Wilms tumor 1 (WT1), a zinc finger protein, and CBP/p300-interacting transactivator 2 (CITED2), which are both expressed at early stages in the urogenital ridge and synergistically promote adrenal development through induction of SF1 transcription (6, 7); pre-B-cell leukemia transcription factor 1 (PBX1), a homeodomain protein, which has been proposed to facilitate the access of important developmental factors (such as SF1) to chromatin to induce differentiation (8, 9); dosage-sensitive sex reversal, adrenal hypoplasia critical region, on chromosome X, gene 1 (DAX1) (10), whose mutations are associated with a variety of developmentally relevant conditions in the adrenal and gonads (11), such as adrenal hypoplasia congenita. Moreover, multiple pathways have been implicated in the fine-tuned regulation of adrenal cortex development and function [reviewed in Ref. (12–15)]. The contribution of each of these signaling pathways has been studied by employing either *in vitro* systems or animal models, such as genetic lineage tracing (16, 17). Not surprisingly, the alteration of some of these pathways, such as the WNT pathway, results in adrenal cancer [reviewed in Ref. (18)].

The adult gland is divided into at least three distinct zones arranged concentrically around the medulla; the zona glomerulosa (ZG, which synthesizes mineralocorticoids), which lies just underneath the capsule, the zona fasciculata (ZF, which synthesizes glucocorticoids), and then the zona reticularis (which synthesizes adrenal androgens in humans and primates). Zonation occurs around birth [for review, see Ref. (12)].

Primary adrenal insufficiency is due to a number of adrenal disorders, and management of these patients can be challenging (19). Current treatments entail lifelong replacement with exogenous steroids. None suitably mimic the diurnal pattern of cortisol noted in healthy individuals, and objective variables to measure quality of replacement are lacking. Fine-tuning of replacement leaves only a narrow margin for improvement. Under-replacement can result in severe impairment of well-being and incipient crisis. Conversely, chronic over-replacement can lead to substantial morbidity including obesity, osteoporosis, and impaired glucose tolerance. Furthermore, despite attention to detail with regards to replacement regimens, overall standardized mortality ratio for patients with adrenal failure is ~2.1.

## Transplantation of Adrenal Glands and Cells

Adrenal autotransplantation in humans is poorly characterized. A few surgeons tested the potential benefits of adrenal autotransplantation in patients with Cushing’s disease since the early 1960s. At that time, replacement therapy with synthetic steroids was becoming the gold standard for patients with Cushing’s disease but they hypothesized that autotransplantation would be an effective alternative to lifelong therapy (20–26). Adrenals were mainly transplanted in the thigh, because it is an easily accessible site should the transplant become not viable and need removal. The degree of autotransplant survival was, however, questionable, at least in those cases where follow-up is available (27). More recently, a successful mother-to-daughter allograft has been reported in a pediatric patient who developed adrenal insufficiency following fulminant meningococcemia (28). Allotransplantations of whole adrenal–kidney or adrenal–kidney–pancreas have also been described in clinical reports (29, 30); however, these multiple organ transplants are only feasible and/or recommended when the recipients have severe co-morbidities.

The refinement of allotransplantation and xenotransplantation has been the focus of more recent research, where a variety of animal models have been employed. Hornsby and colleagues performed initial work, demonstrating that transplantation of human adrenocortical cells into adrenalectomized severe combined immunodeficiency (scid) mice could be an effective technique for the treatment of adrenal insufficiency (31). They also developed a model, in which they showed that co-transplantation in a polycarbonate cylinder of clonal or primary bovine adrenocortical cells with 3T3 fibroblasts into scid mice could rescue them from developing adrenal insufficiency (32, 33). 3T3 cells were either treated with mitomycin C or lethally irradiated (to prevent proliferation upon implantation) and were overexpressing FGF1 (to aid vascularization).

Through their work, they also showed that transplanted adrenocortical cells (of either human or bovine origin) could survive and form a functional, vascularized tissue that was able to replace the host animal’s organ. The newly formed tissue was able to produce and secrete the necessary amounts of cortisol, albeit aldosterone was only produced by primary bovine adrenocortical cells, but not from clonal cells (32). One possible reason for this might be that bovine adrenocortical cells preparations, albeit derived from the ZF, could contain some ZG cells or that some ZF cells could transdifferentiate to a ZG phenotype. It was later shown, however, that ZF cells are incapable of giving rise to ZG cells (34). In the same study, Teebken and Scheumann showed that transplanting ZG cells can give rise to both ZG and ZF, thus making ZG cells more suitable for transplantation (34). This is presumable because the harvest of ZG cells might result in the inclusion of progenitor cells with bi-potency, given their location in the subcapsular region close to fully mature ZG cells in mouse (16, 35) and rat (36, 37). More recently, using mouse cell fate mapping and gene-deletion tools, Freedman and colleagues demonstrated that ZF cells indeed derive from fully differentiated ZG cells through lineage conversion (38). Similar results were observed during adrenal autotransplantation in the spleen of adrenalectomized rats (39).

Furthermore, genetic modifications such as immortalization of bovine primary adrenocortical cultures with telomerase reverse transcriptase (hTERT) have been shown to be very effective in xenotransplantation procedures. This technique aids the expansion of adrenocortical cells *in vitro* leading to improved efficacy (40, 41). Although hTERT is known to be a key-tumorigenic factor, when expressed on its own it did not alter the properties of the donor’s adrenocortical cell (42).

## Possible Platforms for Cell Transplantation

Other groups looked into harnessing the properties of the adrenal cortex extracellular matrix (ECM). It is well established that the ECM plays a significant role in organ growth and development, as well as function, by providing structural support and by regulating cell signaling and cell-to-cell interactions (43). The ECM of the adrenal cortex is quite complex, mainly composed of collagen IV, fibronectin, and laminin and it was postulated that different ECM components might affect the biological activity of cortical cells (44). In fact, primary cultures of human fetal adrenal cells seeded on collagen IV or laminin were found to be more proliferative, as opposed to fibronectin, which enhanced cell apoptosis. In addition, collagen increased cell sensitivity to ACTH, whereas fibronectin and laminin had the opposite effect (45).

Further work to assess the function of implanted cells seeded on a collagen matrix, using a mouse model with adrenal insufficiency, determined that treatment involving staged bilateral adrenalectomy of the animals and implantation of collagen sponges each time was the most beneficial. This protocol lead to a 100% survival rate (up from 42% in mice with only one implant and 0% in animal receiving the graft without cells) and reversal of adrenal insufficiency, supported by restoration of corticosterone levels and the expression of adrenal markers by the transplanted cells (46).

Some researchers focused on the use of the native organ’s own decellularized ECM to act as a scaffold in transplantation procedures to regenerate various organs, including the heart, liver, and lungs (47). *In vitro* studies, using porcine decellularized adrenal ECM as a scaffold for human fetal adrenal cells, resulted in successful cell attachment and a good cell function, the latter assessed by cell proliferation and cortisol secretion (48). Therefore, decellularized adrenal ECM could represent an improved scaffold for transplanted cells, providing a native three-dimensional structure that would support growth and incorporation of transplanted cells into the native environment to form a fully functional adrenal tissue.

## Stem Cells and Reprograming Strategies

An ever-increasing number of translational scientists are striving to take advantage of the remarkable properties of stem cells that are not only offering an unparalleled opportunity to study human biology and disease processes *in vitro* but also to translate basic scientific discoveries to therapeutic applications. The development of cell therapy has, however, been overlooked to date in field of endocrinology, with the exception of the worldwide effort to generate functional pancreatic beta cells to cure type-I diabetes. Indeed, the Californian company Viacyte has been granted FDA approval to launch the first clinical evaluation of a stem-cell-derived islet replacement therapy for the treatment of patients with type-I diabetes in 2015 (http://viacyte.com/clinical/clinical-trials/). Other clinical trials are in the pipeline, a culmination of decades of intense *in vitro* and animal studies carried out by dozens of laboratories (49). Despite the fact that the morbidity of diabetes vs. adrenal insufficiency is clearly not comparable, conceivably the search for alternative treatments for adrenal insufficiency has been neglected, except for a few important discoveries (see below).

Cellular reprograming describes the process where a fully differentiated, specialized cell type is induced to transform into a different cell type that it would not otherwise become under normal physiological conditions. Sir John Gurdon of Britain and Shinya Yamanaka of Japan were awarded the Nobel Prize in 2012 for their groundbreaking discoveries in the field. Gurdon’s research showed that it was possible to reverse the specialization of cells. By transferring a nucleus from a frog’s intestinal cell into a frog’s egg cell that had its nucleus removed, he was able to obtain a tadpole. Building on Gurdon’s work, Yamanaka published a paper in 2006 demonstrating that mature murine cells can become immature stem cells (called inducible pluripotent stem cells, IPSCs) by expressing genes encoding four transcription factors (50). IPSCs can then be differentiated to several tissues using specific cocktails of growth factors/cytokines/chemical compounds. Yamanaka’s breakthrough opened the door to studying tissue-specific diseases and developing diagnosis and treatments. Reprograming is not only achieved through the generation of IPSCs but also through direct reprograming (also known as lineage conversion). Lineage conversion, which is usually achieved by forced expression of lineage-determining factors, is a recently developed and attractive alternative to obtain cells of a given lineage [reviewed in Ref. (51, 52)]. Lineage conversion into several clinically relevant cell types might also prove to be a safer alternative to IPSCs; in fact, genetic (copy number variation, chromosome duplication) and epigenetic variations, which have been described in IPSCs lines and that have raised a number of questions regarding the functional relevance as well as patient safety in potential translational applications, are uncommon during lineage conversion (51).

In recent years, several studies have shown the possibility of obtaining cells with steroidogenic properties resembling adrenocortical cells from murine and human cell sources (Table 1). Pioneering studies in mouse embryonic stem cells (ESCs) showed that the ectopically stable expression of SF1 in the presence of cAMP resulted in a dramatic change of cell morphology and subsequent upregulation of *Cyp11a1* together with an induction of steroidogenesis. However, these cells were incapable of producing different steroid hormones *de novo*, since only progesterone was detected after treatment with 20α-hydroxycholesterol, a freely diffusible form of cholesterol (53).

**Table 1:** **Details of published studies on adrenocortical or adrenogonwadal reprograming**.

PMID	Article	Cells	Origin	Methodology
9199334	Crawford PA, et al. *Mol Cell Biol* (1997)	Embryonic stem cells (ESC)	Mouse (RW4 129/SvJ)	Stable transfection of SF1
15569155	Gondo S, et al. *Genes Cells* (2004)	Bone marrow stem cells (BMCs)	Mouse [C57BL/6Tg14 (act-EGFP)osbY01]	Adenovirus SF1
16728492	Yazawa T, et al. *Endocrinology* (2006)	Bone marrow stem cells (BMCs)	Human (hMSChTERT-E6/E7)	Stable transfection of SF1
17975261	Tanaka T, et al. *J Mol Endocrinol* (2007)	Bone marrow stem cells (BMCs)	Human (commercial cell line)	Adenovirus SF1
18566117	Gondo S, et al. Endocrinology (2008)	Adipose mesenchymal cells (AMCs)	Mouse (C57BL/6J) (B6)	Adenovirus SF1
19359379	Yazawa T, et al. *Endocrinology* (2009)	Bone marrow stem cells (BMCs)	Human (hMSChTERT-E6/E7)	Retrovirus SF1/LRH-1
20133449	Yazawa T, et al. *Mol Endocrinol* (2010)	Umbilical cord blood (UCB-MSCs)	Human (umbilical cord blood)	Retrovirus SF1
21129436	Yazawa T, et al. *Mol Cell Endocrinol* (2011)	Embryonic stem cells (ESC)	Mouse (EBRTcH3)	Retrovirus (inducible SF1)
21610156	Jadhav U, et al. *Endocrinology* (2011)	Embryonic stem cells (ESC)	Mouse (R1 ES cell line)	Stable transfection of SF1
21764617	Mazilu JK, et al. *Mol Genet Metab* (2011)	Mesoderm-derived cells	Human	Adenoviral SF1/Dax1/Cited2/Pbx1/WT1
22324479	Wei X, et al. *Cell Prolif* (2012)	Umbilical cord mesenchymal stem cells (UC-MSCs)	Human (umbilical cord)	Adenovirus SF1
22778223	Sonoyama T, et al. *Endocrinology* (2012)	Embryonic Stem cell (ESC) iPS (from fibroblasts)	Human (H9 and KhES1) human (201B7)	Mesoderm diff. and nucleofection SF1

In 2004, Gondo and colleagues described the capacity of ­long-term cultured mouse bone marrow cells (BMCs) to differentiate into a steroidogenic lineage using adenoviral-mediated SF1 overexpression (54). In this study, the authors were able to detect several steroidogenic enzymes and quantify the levels of all steroid hormones except aldosterone; further study showed that the cells failed to express *Cyp11b2*. Interestingly, reprogramed cells were responsive to ACTH, resulting in enhanced steroidogenic enzyme upregulation and hormone production. Despite the improvement in the steroidogenic profiling compared with the previous study, these cells showed a mixed pattern of adrenal and gonadal phenotypes. Moreover, only supraphysiological concentrations of ACTH were able to significantly induce progesterone and deoxycortisone secretion. It also raises the question of the function of MC2R in BMCs physiology, as the authors found the receptor to be expressed in non-reprogramed cells.

The same group later obtained steroidogenic cells from mouse adipose tissue-derived mesenchymal cells (AMCs), using a similar strategy (55). Remarkably, when the steroidogenic gene expression profile and hormone production of AMCs and BMCs were compared, AMCs showed an enhanced cortisol/testosterone ratio compared with BMCs. This enrichment of the adrenocortical vs. gonadal phenotype was further potentiated by treatment with all-trans retinoic acid (ATRA), suggesting that the tissue/cell source and the culture conditions are essential to determine the resultant phenotype during reprograming.

The first studies describing the generation of steroidogenic cells from human origin were from Miyamoto’s research group. In this work, human mesenchymal stem cells (hMSCs) stably expressing SF1 (as well as hTERT, E6, and E7) were able to upregulate steroidogenic enzymes and produce both adrenal and gonadal steroids after treatment with cAMP. Immunohistochemical analysis of rat GFP^+^ BMCs injected into rat testes showed upregulation of steroidogenic enzymes in the engrafted cells (56). Taken together, these studies demonstrated that MSCs have the ability to differentiate into steroidogenic cells both *in vivo* and *in vitro*.

Since then, several groups have succeeded in differentiating human cells to a steroidogenic phenotype. Tanaka et al. efficiently reprogramed human BMCs upon overexpression of SF1 with adenovirus (57) and more recently this was achieved by retroviral overexpression of LRH-1 [like SF1, a member of the NR5A nuclear receptor family (58)]. However, it is likely that LRH-1 has a more prominent role in gonadal steroidogenesis *in vivo*, since LRH-1 is barely detectable in the adrenal cortex but highly expressed in the gonads, while SF1 is abundantly expressed in both adrenals and gonads (59, 60), and actually at higher levels in cells of the AGP destined to form the adrenal cortex (7).

Human steroidogenic-like cells were also successfully reprogramed from umbilical cord blood mesenchymal stem cells (UCB-MSCs) (61) and umbilical cord Wharton’s jelly-derived MSC (UC-MSCs) (62) through retroviral or adenoviral overexpression of SF1. In the latter study, the authors compared UC-MSCs with BM-MSCs and concluded that UCS-MSCs are the better cell sources since, after reprograming, these cells had a higher proliferative potential, expressed higher levels of steroidogenic enzymes, secreted more steroid hormones, and had a significantly higher cell viability.

Recently, several groups have changed their focus onto ESCs, as the first attempts to reprogram these cells were only partially successful (53). Yazawa et al. reported that to efficiently reprogram mouse ESCs (mESCs) to a steroidogenic phenotype, an initial differentiation to a mesenchymal lineage is needed (63). Once differentiation was achieved (using pulse exposures to ATRA and plating cells into collagen IV-coated dishes), cells became steroidogenic upon overexpression of SF1, resulting in an upregulation of steroidogenic enzymes as well as steroid hormone production. Interestingly, the gene expression profile of these cells was similar to that of the zona fasciculata. Alternatively, Jadhav and Jameson provided evidence that steroidogenic cells can be produced from mESCs using different protocols, the most efficient involving the withdrawal of leukemia inhibitory factor from the ESC medium followed by treatment with cAMP. Using this protocol, steroid-producing reprogramed ESCs appear to acquire a gonadal-like cell type lineage (64). Again, these works highlight the importance of the reprograming strategy/protocol to obtain a specific adrenal/gonadal-like cell type.

Sonoyama et al. demonstrated the capacity of human ES cells (hESCs) to become steroidogenic (65). Using a similar approach as previously reported (63, 64), after differentiation of hESCs to a mesodermal lineage (in this case using a GSK3β inhibitor) and upon overexpression of SF1 and cAMP treatment, hESCs showed overexpression of steroidogenic enzymes as well as hormone production. The authors also obtained the first steroidogenic cells reprogramed from an IPSC line (201B7, obtained from human fibroblasts).

Despite none of the strategies described above have been tested in *in vivo* models of adrenal insufficiency (i.e., adrenalectomized animals), they provide strong evidence that cells can be reprogramed to a steroidogenic phenotype through overexpression of SF1 and cAMP treatment. The choice of the most appropriate source of cells as substrates for reprograming is still debated and might differ depending on downstream applications. Differences in species, cell/tissue source, cellular development stage, epigenetic landscape, SF1/transcription factors dosage (66), regulatory feedbacks of activators/repressors, culture conditions, and timings of reprograming might affect the final phenotype of the reprogramed cells. In this regard, one cannot rule out the possibility that in order to generate fully functional adrenocortical cells, other transcriptions factors might be needed. Factors, such as Dax1, Pbx1, Cited2, WT1, or WNT4, known to be associated with adrenal and gonadal development have been used in reprograming strategies with minor effects on steroidogenic outcome (57, 58, 67). However, given the importance of dosage and regulatory feedback between these key transcription factors, reprograming strategies using a combination of them at specific dosages might be indispensible to obtain cells with a steroidogenic pattern resembling the one found *in vivo*.

There are still several hurdles to overcome to efficiently reprogram human cells to be used for personalized cell-based therapies in patients with adrenal insufficiency in clinics: (1) the conditions to obtain cells with restricted adrenocortical-specific gene expression and hormone production are far from optimized (see above). (2) In most of the protocols, upon overexpression of SF1, cells terminally differentiate with a consequent growth arrest. This makes it difficult to prepare the large amount of cells needed for cell therapy. The use of inducible vectors should allow the expression of SF1 (and/or other factors) at a desired culture time-point when there are enough cells for clinical purposes. (3) The methodologies used until now are all based on the overexpression of SF1 exogenously, either episomally or virally. Optimization of the culture conditions in a gene delivery-free model of reprograming might help to avoid the safety concerns about these cells. The use of excisable vectors together with the new CRISPR-dCasVP64 technology to activate specific transgenes without the use of transcription factors (68) might reduce the safety concerns of this method of obtaining reprogramed steroidogenic cells *in vitro*.

## Recent Breakthroughs in Overcoming Rejection and the Treatment of Monogenic Diseases

A major factor limiting the application of stem-cell therapy in patients transplanted with ECs-derived adrenocortical cells or even autologous IPSCs-derived adrenocortical cells (i.e., in those individuals affected by autoimmune Addison’s) is the recipient’s need to adhere to lifelong immunosuppression. An effort going back a couple of decades in the field of biomaterials is providing us with the technology, which can make an impact in the clinical setting today; in fact, several encapsulation devices (endowed with excellent biocompatibility) have been developed and are undergoing clinical testing in patients with type-I diabetes. Animal studies (49) and published preliminary human studies (69) have demonstrated that the device’s semipermeable membranes can tightly immune isolate transplanted cells while allowing diffusion of nutrients, such as glucose. Therefore, encapsulating adrenocortical cells is a strategy that should prevent rejection of the grafted tissue, whichever the source of it.

In recent times, a fast-paced development of gene-editing technologies using different approaches has made it possible to correct known disease-causing mutations without leaving any footprint (70). This technology could be successfully applicable to monogenic conditions causing adrenal insufficiency (such as congenital adrenal hyperplasia or familial glucocorticoid deficiency). For example, cultures of reprogrammable cell sources such as skin fibroblasts or urine-derived cells could be established and the mutated gene reverted to a wild-type status via gene editing; after successful reprograming to an adrenocortical phenotype (via IPSCs generation or via lineage conversion), cells could be reimplanted back into the donor, housed either inside an encapsulation device or a decellularized adrenal gland obtained from cadaver or large animal.

**Figure 1:**
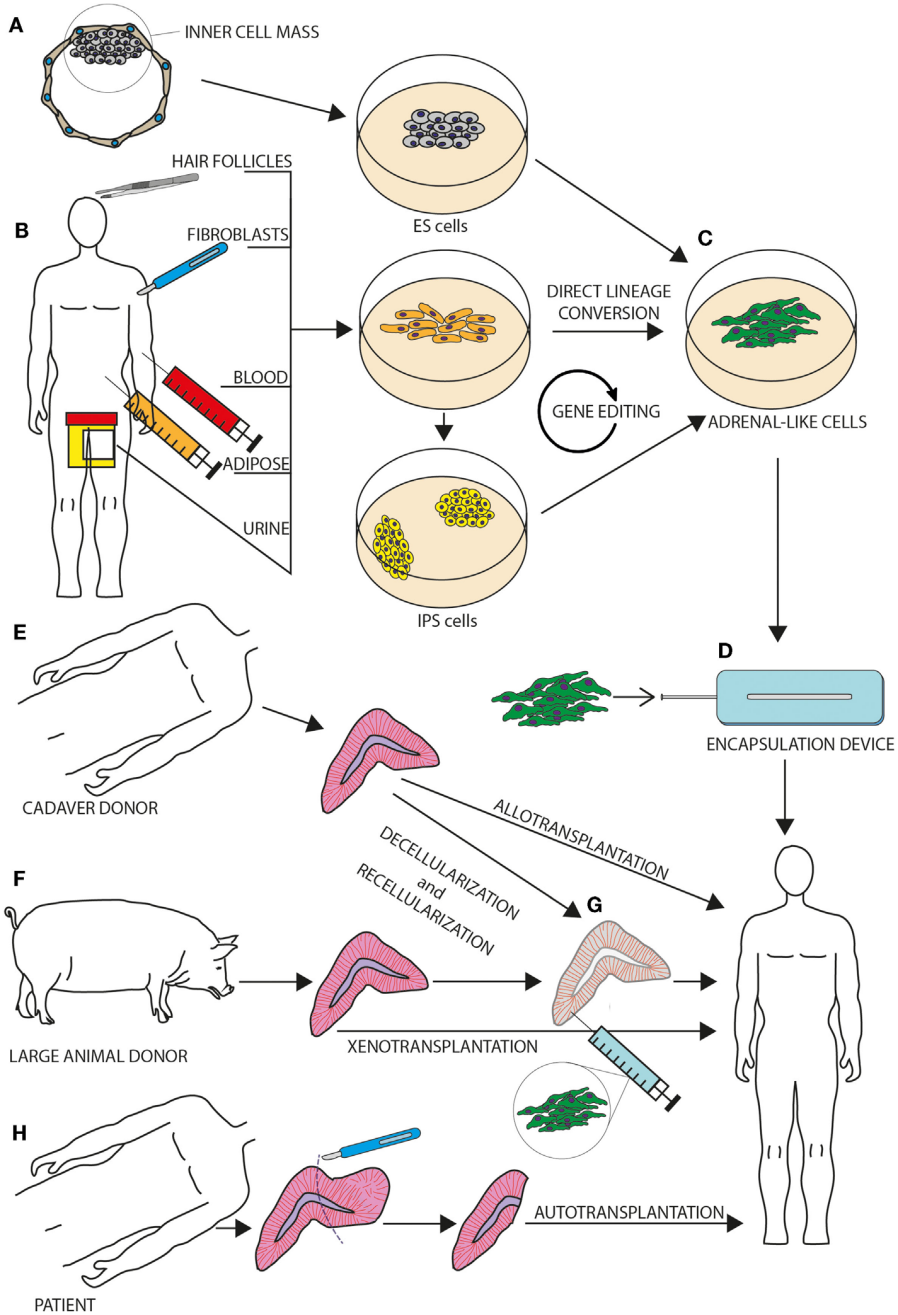
**This figure outlines novel and tested strategies for the treatment of adrenal insufficiency**. Human embryonic stem (ES) cells **(A)** as well as somatic cells (such as fibroblasts, hair follicle dermal papillae, adipose tissue-derived stem cells, and urine-derived stem cells) established from human donors **(B)** can be cultured *in vitro* and induced to acquire an adrenocortical phenotype **(C)** through specific differentiation protocols. A gene-editing step can be included in case of monogenic disorders. Reprogramed cells that have successfully acquired an adrenocortical phenotype could then be implanted back into a donor, either inside an encapsulation device **(D)** or inside a decellularized adrenal of human or large-animal origin **(G)**. While autotransplantation **(H)** has been trailed in humans in pioneering surgery during the 1960s (see text) and allotransplantation **(E)** has been a poorly tested option, xenotransplantation **(F)** has never been tested in humans.

Potential future strategies, as well as pioneering past and present attempts to cure adrenal insufficiency are outlined in Figure 1.

In conclusion, while autotransplantation has long been discarded, allotransplantation is currently being considered only in specific clinical settings and xenotransplantation has not reached the bedside, stem-cell biology has granted great promise for tissue engineering and regenerative medicine. Treatments employing stem cells offer the potential to successfully treat patients with new modalities in the future, by essentially regenerating and replacing non-functional tissues or organs. A permanent collaborative and multidisciplinary effort carried out by translational scientists with expertise in stem cells, bioengineering, and material science is undeniably necessary to fully develop alternative treatments for adrenal insufficiency that have true clinical relevance.

## Conflict of Interest Statement

The authors declare that the research was conducted in the absence of any commercial or financial relationships that could be construed as a potential conflict of interest.

## References

[1] Hatano O Takakusu A Nomura M Morohashi K. Identical origin of adrenal cortex and gonad revealed by expression profiles of Ad4BP/SF-1. Genes Cells (1996) 1(7):663–71. 10.1046/j.1365-2443.1996.00254.x 9078392

[2] Luo X Ikeda Y Parker KL. A cell-specific nuclear receptor is essential for adrenal and gonadal development and sexual differentiation. Cell (1994) 77(4):481–90. 10.1016/0092-8674(94)90211-9 8187173

[3] Schimmer BP White PC. Minireview: steroidogenic factor 1: its roles in differentiation, development, and disease. Mol Endocrinol (2010) 24(7):1322–37. 10.1210/me.2009-0519 20203099 PMC5417463

[4] Wong M Ikeda Y Luo X Caron KM Weber TJ Swain A Steroidogenic factor 1 plays multiple roles in endocrine development and function. Recent Prog Horm Res (1997) 52:167–82. 9238852

[5] Buaas FW Gardiner JR Clayton S Val P Swain A. In vivo evidence for the crucial role of SF1 in steroid-producing cells of the testis, ovary and adrenal gland. Development (2012) 139(24):4561–70. 10.1242/dev.087247 23136395 PMC3509722

[6] Wilhelm D Englert C. The Wilms tumor suppressor WT1 regulates early gonad development by activation of Sf1. Genes Dev (2002) 16(14):1839–51. 10.1101/gad.220102 12130543 PMC186395

[7] Val P Martinez-Barbera JP Swain A. Adrenal development is initiated by Cited2 and Wt1 through modulation of Sf-1 dosage. Development (2007) 134(12):2349–58. 10.1242/dev.004390 17537799

[8] Schnabel CA Selleri L Cleary ML. Pbx1 is essential for adrenal development and urogenital differentiation. Genesis (2003) 37(3):123–30. 10.1002/gene.10235 14595835

[9] Berkes CA Bergstrom DA Penn BH Seaver KJ Knoepfler PS Tapscott SJ. Pbx marks genes for activation by MyoD indicating a role for a homeodomain protein in establishing myogenic potential. Mol Cell (2004) 14(4):465–77. 10.1016/S1097-2765(04)00260-6 15149596

[10] Clipsham R McCabe ER. DAX1 and its network partners: exploring complexity in development. Mol Genet Metab (2003) 80(1–2):81–120. 10.1016/j.ymgme.2003.08.023 14567960

[11] El-Khairi R Martinez-Aguayo A Ferraz-de-Souza B Lin L Achermann JC. Role of DAX-1 (NR0B1) and steroidogenic factor-1 (NR5A1) in human adrenal function. Endocr Dev (2011) 20:38–46. 10.1159/000321213 21164257

[12] Kim AC Barlaskar FM Heaton JH Else T Kelly VR Krill KT In search of adrenocortical stem and progenitor cells. Endocr Rev (2009) 30(3):241–63. 10.1210/er.2008-0039 19403887 PMC2726842

[13] Walczak EM Hammer GD. Regulation of the adrenocortical stem cell niche: implications for disease. Nat Rev Endocrinol (2015) 11(1):14–28. 10.1038/nrendo.2014.166 25287283 PMC4648246

[14] Laufer E Kesper D Vortkamp A King P Sonic hedgehog signaling during adrenal development. Mol Cell Endocrinol (2012) 351(1):19–27 10.1016/j.mce.2011.10.002 22020162 PMC3288303

[15] Gallo-Payet N Battista MC. Steroidogenesis-adrenal cell signal transduction. Compr Physiol (2014) 4(3):889–964. 10.1002/cphy.c130050 24944026

[16] King P Paul A Laufer E. Shh signaling regulates adrenocortical development and identifies progenitors of steroidogenic lineages. Proc Natl Acad Sci U S A (2009) 106(50):21185–90. 10.1073/pnas.0909471106 19955443 PMC2786892

[17] Walczak EM Kuick R Finco I Bohin N Hrycaj SM Wellik DM Wnt signaling inhibits adrenal steroidogenesis by cell-autonomous and non-cell-autonomous mechanisms. Mol Endocrinol (2014) 28(9):1471–86. 10.1210/me.2014-1060 25029241 PMC4154239

[18] Else T Kim AC Sabolch A Raymond VM Kandathil A Caoili EM Adrenocortical carcinoma. Endocr Rev (2014) 35(2):282–326. 10.1210/er.2013-1029 24423978 PMC3963263

[19] Bancos I Hahner S Tomlinson J Arlt W Diagnosis and management of adrenal insufficiency. Lancet Diabetes Endocrinol (2015) 3(3):216–26 10.1016/S2213-8587(14)70142-1 25098712

[20] Hardy JD. Surgical management of Cushing’s syndrome with emphasis on adrenal autotransplantation. Ann Surg (1978) 188(3):290–307. 10.1097/00000658-197809000-00004 686895 PMC1396962

[21] Hardy JD Autotransplantation of adrenal remnant to high in Cushing’s disease. Preserving residual cortical activity while avoiding laparotomy. JAMA (1963) 185:134–6 10.1001/jama.1963.03060020094036 13960766

[22] Hardy JD Langford HG Adrenal autotransplantation in Cushing’s disease. Ann N Y Acad Sci (1964) 120:667–8 10.1111/j.1749-6632.1965.tb30692.x 14235281

[23] Ibbertson HK O’Brien KP Adrenal autografts in treatment of Cushing’s disease. Br Med J (1962) 2(5306):703–6 10.1136/bmj.2.5306.703 14450493 PMC1925991

[24] Franksson C Birke G Plantin LO Adrenal autotransplantation in Cushing’s syndrome. Acta Chir Scand (1959) 117:409–15. 13824587

[25] Birke G Franksson C Moberger G Plantin LO Storage and autotransplantation of human adrenal tissue. Acta Chir Scand (1956) 111(2):113–23. 13354217

[26] Drucker WD Localio SA Becker MH Bergman B Autotransplantation of hyperplastic human adrenal tissue. Arch Intern Med (1967) 120(2):185–92 10.1001/archinte.1967.00300020057007 4952672

[27] Hardy JD Moore DO Langford HG. Cushing’s disease today. Late follow-up of 17 adrenalectomy patients with emphasis on eight with adrenal autotransplants. Ann Surg (1985) 201(5):595–603. 10.1097/00000658-198505000-00008 2986564 PMC1250767

[28] Grodstein E Hardy MA Goldstein MJ. A case of human intramuscular adrenal gland transplantation as a cure for chronic adrenal insufficiency. Am J Transplant (2010) 10(2):431–3. 10.1111/j.1600-6143.2009.02929.x 19958326

[29] Vouillarmet J Buron F Houzard C Carlier MC Chauvet C Brunet M The first simultaneous kidney-adrenal gland-pancreas transplantation: outcome at 1 year. Am J Transplant (2013) 13(7):1905–9. 10.1111/ajt.12296 23731324

[30] Dubernard JM Cloix P Tajra LC Alduglihan W Borson F Lefrancois N Simultaneous adrenal gland and kidney allotransplantation after synchronous bilateral renal cell carcinoma: a case report. Transplant Proc (1995) 27(1):1320–1. 7878899

[31] Thomas M Northrup SR Hornsby PJ. Adrenocortical tissue formed by transplantation of normal clones of bovine adrenocortical cells in scid mice replaces the essential functions of the animals’ adrenal glands. Nature (1997) 3(9):978–83. 9288723 10.1038/nm0997-978

[32] Thomas M Hornsby PJ. Transplantation of primary bovine adrenocortical cells into scid mice. Mol Cell Endocrinol (1999) 153:125–36. 10.1016/S0303-7207(99)00070-2 10459860

[33] Thomas M Wang X Hornsby PJ. Human adrenocortical cell xenotransplantation: model of cotransplantation of human adrenocortical cells and 3T3 cells in scid mice to form vascularized tissue and prevent adrenal insufficiency. Xenotransplantation (2002) 9:58–67. 10.1046/j.0908-665x.2001.00138.x 12005105

[34] Teebken OE Scheumann GFW. Differentiated corticosteroid production and regeneration after selective transplantation of cultured and noncultured adrenocortical cells in the adrenalectomized rat. Transplantation (2000) 70(5):836–43. 10.1097/00007890-200009150-00022 11003367

[35] Huang CC Miyagawa S Matsumaru D Parker KL Yao HH. Progenitor cell expansion and organ size of mouse adrenal is regulated by sonic hedgehog. Endocrinology (2010) 151(3):1119–28. 10.1210/en.2009-0814 20118198 PMC2840682

[36] Guasti L Paul A Laufer E King P. Localization of Sonic hedgehog secreting and receiving cells in the developing and adult rat adrenal cortex. Mol Cell Endocrinol (2011) 336(1–2):117–22. 10.1016/j.mce.2010.11.010 21094676 PMC3063526

[37] Guasti L Candy Sze WC McKay T Grose R King PJ. FGF signalling through Fgfr2 isoform IIIb regulates adrenal cortex development. Mol Cell Endocrinol (2013) 371(1–2):182–8. 10.1016/j.mce.2013.01.014 23376610 PMC3650577

[38] Freedman BD Kempna PB Carlone DL Shah MS Guagliardo NA Barrett PQ Adrenocortical zonation results from lineage conversion of differentiated zona glomerulosa cells. Dev Cell (2013) 26(6):666–73. 10.1016/j.devcel.2013.07.016 24035414 PMC3791142

[39] Allende G Chavira R Quintanar-Stephano A. Biochemical evidence of the functional recovery and regeneration of adrenal autotransplants in the rat spleen. Endocrine (2001) 16(3):173–9. 10.1385/ENDO:16:3:173 11954660

[40] Thomas M Yang L Hornsby PJ. Formation of functional tissue from transplanted adrenocortical cells expressing telomerase reverse transcriptase. Nat Biotechnol (2000) 18:39–42. 10.1038/71894 10625388

[41] Huang Q Chen M Liang S Acha V Liu D Yuan F Improving cell therapy – experiments using transplanted telomerase-immortalized cells in immunodeficient mice. Mech Ageing Dev (2007) 128(1):25–30. 10.1016/j.mad.2006.11.006 17123586 PMC1797893

[42] Thomas M Suwa T Yang L Zhao L Hawks CL Hornsby PJ. Cooperation of hTERT, SV40 T Antigen and oncogenic Ras in tumorigenesis: a cell transplantation model using bovine adrenocortical cells. Neoplasia (2002) 4(6):493–500. 10.1038/sj.neo.7900262 12407443 PMC1503663

[43] Badylak SF. The extracellular matrix as a scaffold for tissue reconstruction. Cell Dev Biol (2002) 13:377–83. 10.1016/S1084952102000940 12324220

[44] Chamoux E Bolduc L Lehoux JG Gallo-Payet N. Identification of extracellular matrix components and their integrin receptors in the human fetal adrenal gland. J Clin Endocrinol Metab (2001) 86(5):2090–8. 10.1210/jcem.86.5.7462 11344212

[45] Chamoux E Narcy A Lehoux J-G Gallo-Payet N. Fibronectin, laminin and collagen IV as modulators of cell behaviour during adrenal gland development in the human fetus. J Clin Endocrinol Metab (2002) 87(4):1819–28. 10.1210/jcem.87.4.8359 11932324

[46] Zupekan T Dunn JCY. Adrenocortical cell transplantation reverses a murine model of adrenal failure. J Pediatr Surg (2011) 46(6):1208–13. 10.1016/j.jpedsurg.2011.03.057 21683224 PMC3118992

[47] Faulk DM Johnson SA Zhang L Badylak SF. Role of the extracellular matrix in whole organ engineering. J Cell Physiol (2014) 229(8):984–9. 10.1002/jcp.24532 24347365

[48] Allen RA Seltz LM Jiang BSH Kasick RT Sellaro TL Badylak SF Adrenal extracellular matrix scaffolds support adrenocortical cell proliferation and function in vitro. Tissue Eng (2010) 16(11):3363–74. 10.1089/ten.TEA.2010.0005 20528677

[49] Cogger K Nostro MC Recent advances in cell replacement therapies for the treatment of type 1 diabetes. Endocrinology (2015) 156(1):8–15 10.1210/en.2014-1691 25386833

[50] Takahashi K Yamanaka S. Induction of pluripotent stem cells from mouse embryonic and adult fibroblast cultures by defined factors. Cell (2006) 126(4):663–76. 10.1016/j.cell.2006.07.024 16904174

[51] Vierbuchen T Wernig M. Molecular roadblocks for cellular reprogramming. Mol Cell (2012) 47(6):827–38. 10.1016/j.molcel.2012.09.008 23020854 PMC3809030

[52] Sancho-Martinez I Baek SH Izpisua Belmonte JC. Lineage conversion methodologies meet the reprogramming toolbox. Nat Cell Biol (2012) 14(9):892–9. 10.1038/ncb2567 22945254

[53] Crawford PA Sadovsky Y Milbrandt J. Nuclear receptor steroidogenic factor 1 directs embryonic stem cells toward the steroidogenic lineage. Mol Cell Biol (1997) 17(7):3997–4006. 9199334 10.1128/mcb.17.7.3997 PMC232252

[54] Gondo S Yanase T Okabe T Tanaka T Morinaga H Nomura M SF-1/Ad4BP transforms primary long-term cultured bone marrow cells into ACTH-responsive steroidogenic cells. Genes Cells (2004) 9(12):1239–47. 10.1111/j.1365-2443.2004.00801.x 15569155

[55] Gondo S Okabe T Tanaka T Morinaga H Nomura M Takayanagi R Adipose tissue-derived and bone marrow-derived mesenchymal cells develop into different lineage of steroidogenic cells by forced expression of steroidogenic factor 1. Endocrinology (2008) 149(9):4717–25. 10.1210/en.2007-1808 18566117

[56] Yazawa T Mizutani T Yamada K Kawata H Sekiguchi T Yoshino M Differentiation of adult stem cells derived from bone marrow stroma into Leydig or adrenocortical cells. Endocrinology (2006) 147(9):4104–11. 10.1210/en.2006-0162 16728492

[57] Tanaka T Gondo S Okabe T Ohe K Shirohzu H Morinaga H Steroidogenic factor 1/adrenal 4 binding protein transforms human bone marrow mesenchymal cells into steroidogenic cells. J Mol Endocrinol (2007) 39(5):343–50. 10.1677/JME-07-0076 17975261

[58] Yazawa T Inanoka Y Mizutani T Kuribayashi M Umezawa A Miyamoto K. Liver receptor homolog-1 regulates the transcription of steroidogenic enzymes and induces the differentiation of mesenchymal stem cells into steroidogenic cells. Endocrinology (2009) 150(8):3885–93. 10.1210/en.2008-1310 19359379

[59] Parker KL Schimmer BP Steroidogenic factor 1: a key determinant of endocrine development and function. Endocr Rev (1997) 18(3):361–77 10.1210/edrv.18.3.0301 9183568

[60] Fayard E Auwerx J Schoonjans K. LRH-1: an orphan nuclear receptor involved in development, metabolism and steroidogenesis. Trends Cell Biol (2004) 14(5):250–60. 10.1016/j.tcb.2004.03.008 15130581

[61] Yazawa T Inaoka Y Okada R Mizutani T Yamazaki Y Usami Y PPAR-gamma coactivator-1alpha regulates progesterone production in ovarian granulosa cells with SF-1 and LRH-1. Mol Endocrinol (2010) 24(3):485–96. 10.1210/me.2009-0352 20133449 PMC5419099

[62] Wei X Peng G Zheng S Wu X. Differentiation of umbilical cord mesenchymal stem cells into steroidogenic cells in comparison to bone marrow mesenchymal stem cells. Cell Prolif (2012) 45(2):101–10. 10.1111/j.1365-2184.2012.00809.x 22324479 PMC6496766

[63] Yazawa T Kawabe S Inaoka Y Okada R Mizutani T Imamichi Y Differentiation of mesenchymal stem cells and embryonic stem cells into steroidogenic cells using steroidogenic factor-1 and liver receptor homolog-1. Mol Cell Endocrinol (2011) 336(1–2):127–32. 10.1016/j.mce.2010.11.025 21129436

[64] Jadhav U Jameson JL. Steroidogenic factor-1 (SF-1)-driven differentiation of murine embryonic stem (ES) cells into a gonadal lineage. Endocrinology (2011) 152(7):2870–82. 10.1210/en.2011-0219 21610156 PMC3192422

[65] Sonoyama T Sone M Honda K Taura D Kojima K Inuzuka M Differentiation of human embryonic stem cells and human induced pluripotent stem cells into steroid-producing cells. Endocrinology (2012) 153(9):4336–45. 10.1210/en.2012-1060 22778223

[66] Val P Swain A Gene dosage effects and transcriptional regulation of early mammalian adrenal cortex development. Mol Cell Endocrinol (2010) 323(1):105–14 10.1016/j.mce.2009.12.010 20025938

[67] Mazilu JK McCabe ER. Moving toward personalized cell-based interventions for adrenal cortical disorders: part 2 – human diseases and tissue engineering. Mol Genet Metab (2011) 104(1–2):80–8. 10.1016/j.ymgme.2011.06.011 21764617

[68] Kearns NA Genga RM Enuameh MS Garber M Wolfe SA Maehr R. Cas9 effector-mediated regulation of transcription and differentiation in human pluripotent stem cells. Development (2014) 141(1):219–23. 10.1242/dev.103341 24346702 PMC3865759

[69] Ludwig B Reichel A Steffen A Zimerman B Schally AV Block NL Transplantation of human islets without immunosuppression. Proc Natl Acad Sci U S A (2013) 110(47):19054–8. 10.1073/pnas.1317561110 24167261 PMC3839710

[70] Cox DB Platt RJ Zhang F. Therapeutic genome editing: prospects and challenges. Nat Med (2015) 21(2):121–31. 10.1038/nm.3793 25654603 PMC4492683

